# Supplementary material for: Polarization patterning in ferroelectric nematic liquids via flexoelectric coupling
Source: Nat Commun. 2023 May 25;14:3029. doi: 10.1038/s41467-023-38749-2 (PMC10213025; doi:10.1038/s41467-023-38749-2)
Supplement: Supplementary file 1 — Supplementary Information [file 41467_2023_38749_MOESM1_ESM.pdf]

Supplementary Information

Polarization patterning in ferroelectric nematic liquids via flexoelectric coupling

N. Sebastián[1], M. Lovšin[1,2], B. Berteloot[3], N. Osterman[1,2], A. Petelin[1,2], R. J. Mandle[4,5], S. Aya[6], M. Huang[6], I. Drevenšek-Olenik[1,2], K. Neyts[3], A. Mertelj[1]

1 Jožef Stefan Institute, Ljubljana, Slovenia
2 University of Ljubljana, Faculty of Mathematics and Physics, Ljubljana, Slovenia
3 Liquid Crystals and Photonics Group, ELIS Department, Ghent University, Ghent, Belgium
4 School of Physics and Astronomy, University of Leeds, Leeds, UK
5 School of Chemistry, University of Leeds, Leeds, UK
6 South China Advanced Institute for Soft Matter Science and Technology (AISMST), School of Emergent Soft Matter, South China University of Technology, Guangzhou, China
7 Guangdong Provincial Key Laboratory of Functional and Intelligent Hybrid Materials and Devices, South China University of Technology, Guangzhou, China

Table of contents:

Supplementary note I: Materials and methods

Supplementary note II: Uniform Patterns

Supplementary note III: Periodic Splay patterns

Supplementary note IV: Dtmm transmission spectra simulations

Supplementary note V: Polarization guiding structures

Supplementary note VI: Model for the structure of the splay patterns

Supplementary note VII: Interferograms fitting function

Supplementary Note I – Materials and Methods

DIO: Iso 173 N 83.9 N_S 68.9 N_F 47 Cr

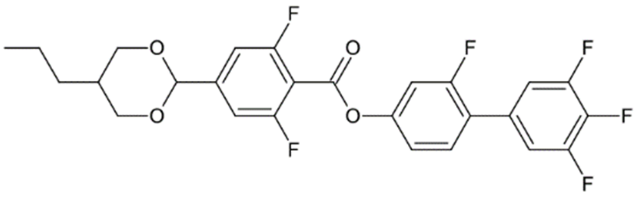

RM734: Iso 187 N 132.7 N_F 90 Cr

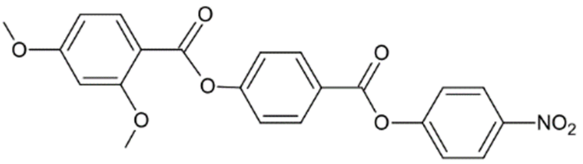

Supplementary Fig.1. Chemical structure of the two materials employed in this work, DIO and RM734, and their phase sequence on cooling[1,2]. While the latter shows on cooling a direct phase transition between the nonpolar nematic phase and the ferroelectric nematic phase, DIO exhibits three nematic phases; on cooling, a high temperature nonpolar nematic phase, followed by an antiferroelectric splay nematic phase and at lower temperatures the ferroelectric nematic phase focus of this work.

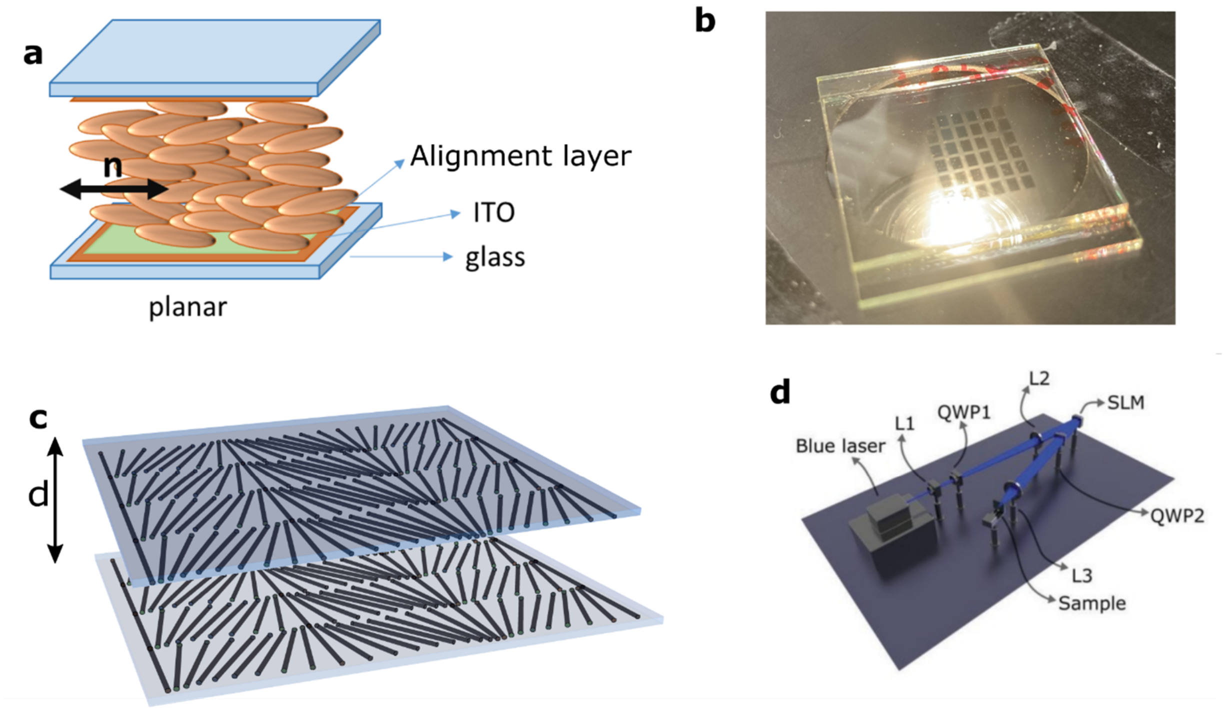

Supplementary Fig.2 Image illustrating typical liquid crystal cells and surface alignment via photopatterning. The described POM, SHG-M and SHG-I observations were done employing home-made glass cells, consisting of two transparent glass plates between which the liquid crystalline material is introduced by capillary action at a temperature of the nematic phase. In our case, both glass plates are lithographed with a transparent conductive layer of Indium Tin Oxide (ITO). Two different electrode geometries were employed, either uniform in both substrates, or interdigitated in one substrate and uniform in the other. In order to prescribe a defined in-plane orientation (planar alignment) of the nematic director, liquid crystal cells additionally incorporate an alignment layer. To achieve well-defined patterned directions of the molecular director at the cell interface, we exploit photoalignment technology, i.e. we can achieve non-uniform liquid crystalline alignment by use of a polarization-sensitive material, which will provide a preferred in-plane (planar) director alignment in a direction perpendicular to the incoming light polarization and illuminating a thin photoalignment layer spin-coated at the surfaces by pattered polarization illumination via a SLM device. a) Schematic representation of a liquid crystalline cell. b) Image of one of the photopatterned cells used in this study, in which the rectangular areas corresponding to different patterns can be clearly seen. c) The image illustrates as an example the alignment pattern at the bottom and top substrates for a periodic splay structure with a maximum angle of 40 degrees. d) Scheme of the photopatterning setup, consisting of a blue laser, a spatial light modulator (SLM), three lenses and quarter wave plates (QWPs), reproduced from reference 3 © 2021 Wiley-VCH GmbH. To achieve the patterned substrates, the cell's photoalignment layers are illuminated with linearly polarized light of which the polarization orientation varies spatially according to the desired orientation of the LC director at the substrates. To do so, the various orientations of linear polarization are generated using a spatial light modulator (SLM, Holoeye Pluto 2), with a resolution of 1920 by 1080 pixels and a pixel pitch of 8 µm. The SLM displays a grayscale image (= the photoalignment pattern) in which each grey level corresponds to a specific orientation of linear polarization. By means of a projection lens, the SLM displayed pattern was scaled down by a factor of 11.6. As a light source a linearly polarized blue laser (Cobolt Twist, λ = 457 nm) was used, which is first transformed into circularly polarized light and reflected via the SLM introducing a phase delay between the vertical and the horizontal field components. Such phase delay determines the azimuthal angle of the linearly polarized light that incises the photoalignment layers.

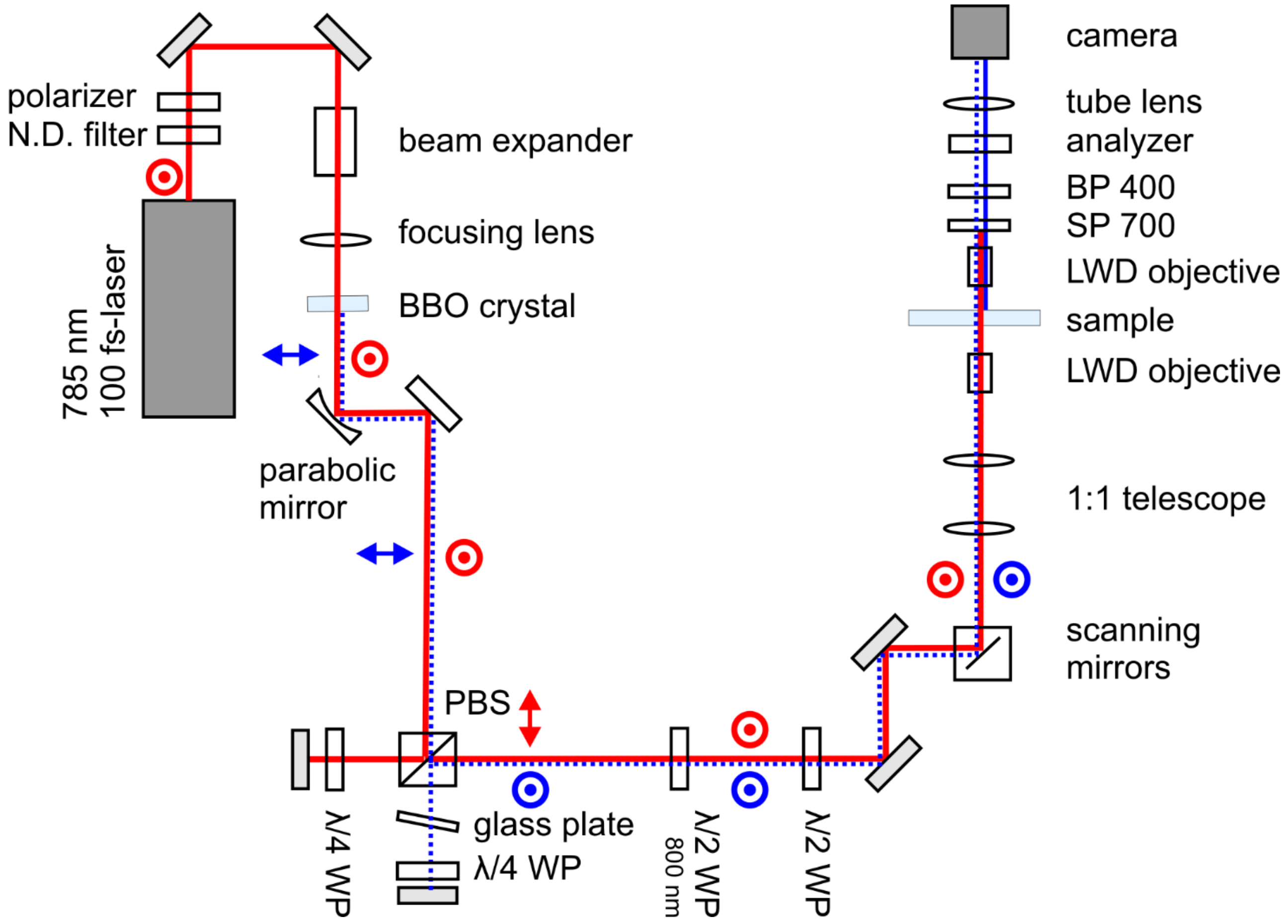

Supplementary Fig.3 Schematics of the Interferometric SHG imaging system as described in the main manuscript in Methods.

Supplementary Note II – Uniform patterns

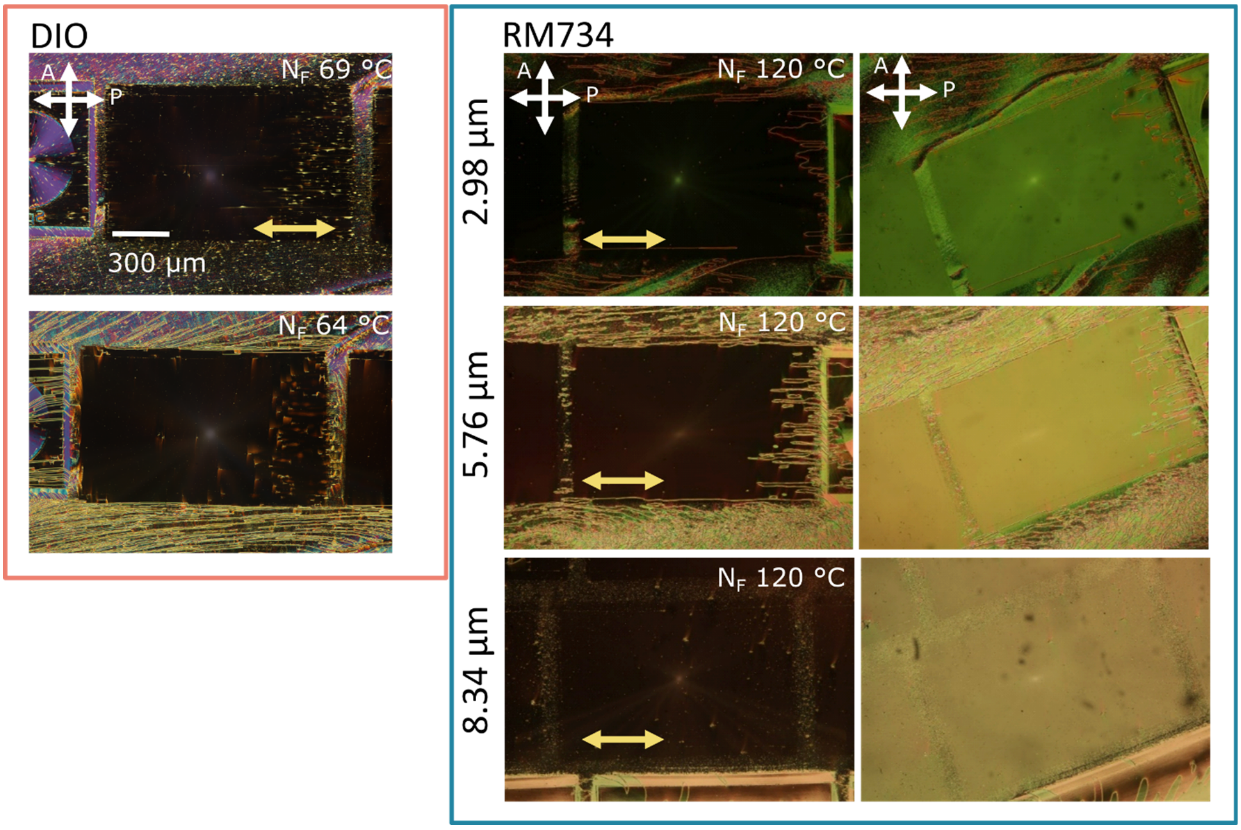

Supplementary Fig.4. (Left) Detail under crossed polarizers of the horizontal pattern and the surroundings for two temperatures in the ferroelectric nematic phase in DIO, before (69 °C) and after (64 °C) what is addressed in the manuscript as wall propagation. Both cell surfaces have a uniform conductive ITO layer. The appearance of domain walls is clearly observed in the surrounding unpatterned background and in the visible corner of the left pattern. The domain wall-free state can be stabilized for around 5 degrees under slow cooling. Cell thickness is 3 µm. (Right) Uniform photopatterned area in cells with different thicknesses d= 2.98, 5.76 and 8.34 µm filled with RM734 at a temperature in the N_F phase (120 °C) showing uniform alignment throughout the 1.3 x 0.7 mm^2 patterned area.

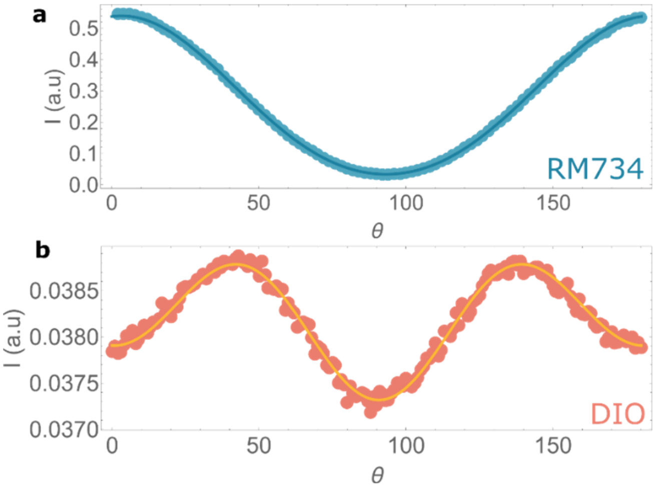

Supplementary Fig.5. Dependence of the recorded SHG intensity in horizontal patterns on the angle θ, measured between the incoming laser polarization and the preferred photoalignment orientation for a) RM734 and b) DIO. Results show for RM734 a maximum SHG signal for incoming polarization along the photoalignment orientation, while for DIO, the maximum is obtained at 45°. Such behaviour evidences that for RM734 the χ_{333} susceptibility coefficient is predominant, while for DIO the χ_{131} coefficient also contributes notably to the signal[4]. The results can be fitted by $A\cos^4(\theta) + B\cos^2(\theta)\sin^2(\theta) + C\sin^4(\theta) + D$.

Supplementary Note III – Periodic Splay patterns

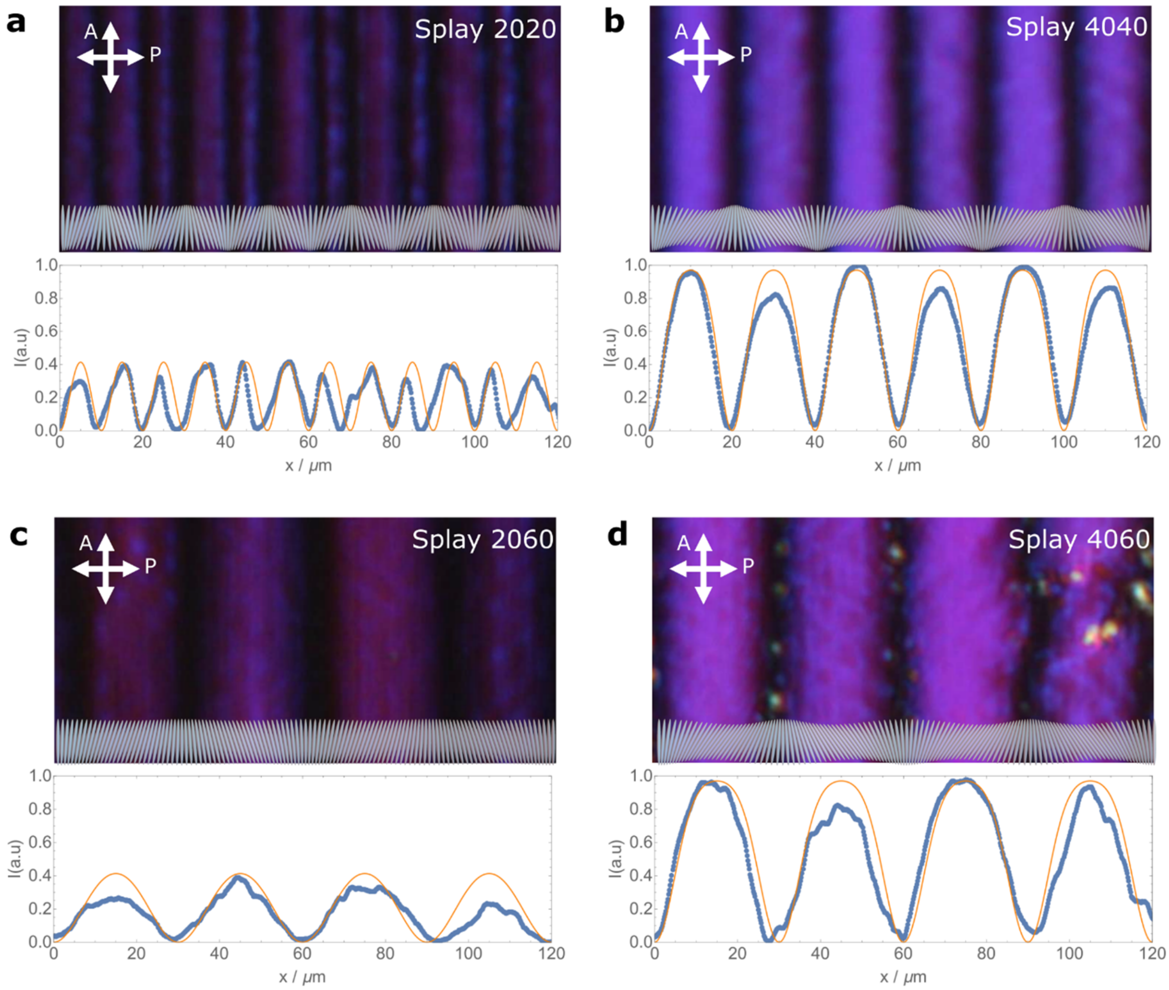

Supplementary Fig.6. Splay pattern profiles in the in the nematic phase 115 ^{0}C of DIO. POM images of 4 different one dimensional splay patterns in which the director deviates from the vertical direction by $\vartheta = \vartheta_0 \sin(2\pi x/P)$, where x corresponds to the horizontal direction, ϑ_0 to the amplitude and P to the period of the pattern are: a) $\vartheta_0 = 20°$, $P = 20\ \mu m$, b) $\vartheta_0 = 40°$, $P = 40\ \mu m$, c) $\vartheta_0 = 20°$, $P = 60\ \mu m$ and d) $\vartheta_0 = 40°$, $P = 60\ \mu m$ and illustrated by the overlaying sketches. Intensity plots show the comparison of the normalized measured intensities across the horizontal direction calculated from the POM images (blue circles) and the theoretical expected intensity profile $I = I_0 \sin(2\phi)^2$ where ϕ is the angle between the prescribed director orientation and the crossed polarizers (orange line).

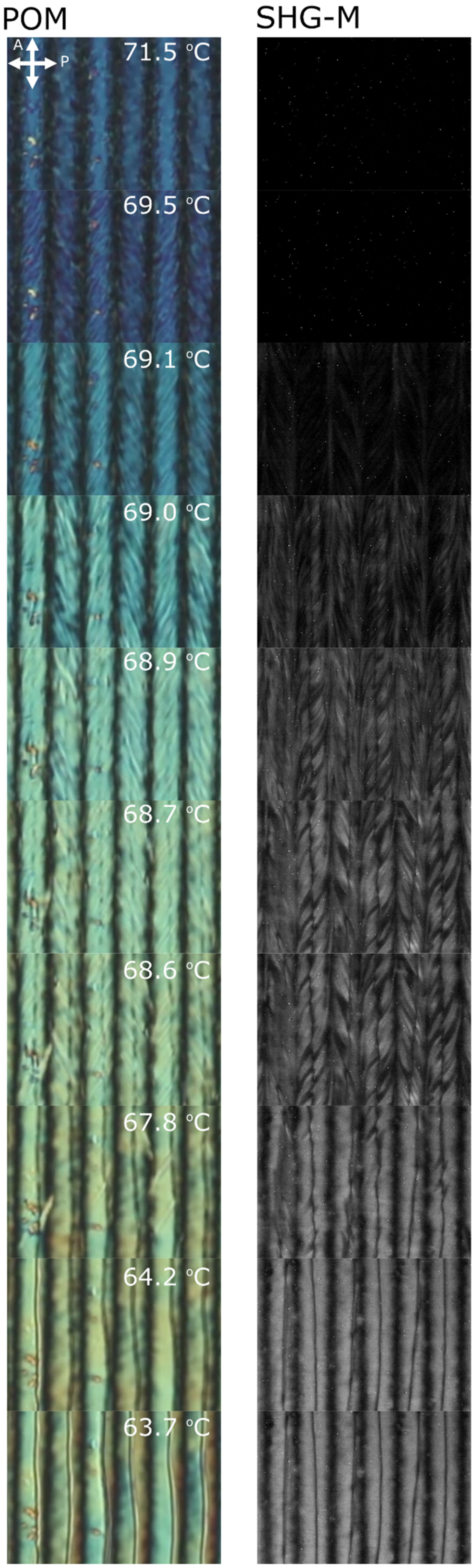

Supplementary Fig.7. Snapshots of the N_S-N_F transition in a periodic Splay pattern with maximum angle of 40 degrees and a period P=40 µm as observed by POM (left) and SHG-M (right) in a 3 µm cell with one of the substrates with an inter-digitated ITO patterned electrode (equivalent to that shown in Fig.1.d in the main manuscript). The texture corresponds to an area without ITO in the inter-digitated electrode, showing that despite the absence of ITO the surface splay prescribed preferred director direction prevents the formation of random positioned domains as shown in Figure 1 in the main manuscript. N_S-N_F pretransitional behaviour is evidenced by the stripe texture appearing overlaid on the prescribed pattern. Disclination lines appear smoothly through the transition along the full pattern in those areas where the splay changes sign (see for example temperatures 68.9 °C and 68.7 °C). Final structural relaxation can be observed in the last POM image of the sequence. On SHG-M the changes associated to it are neglectable.

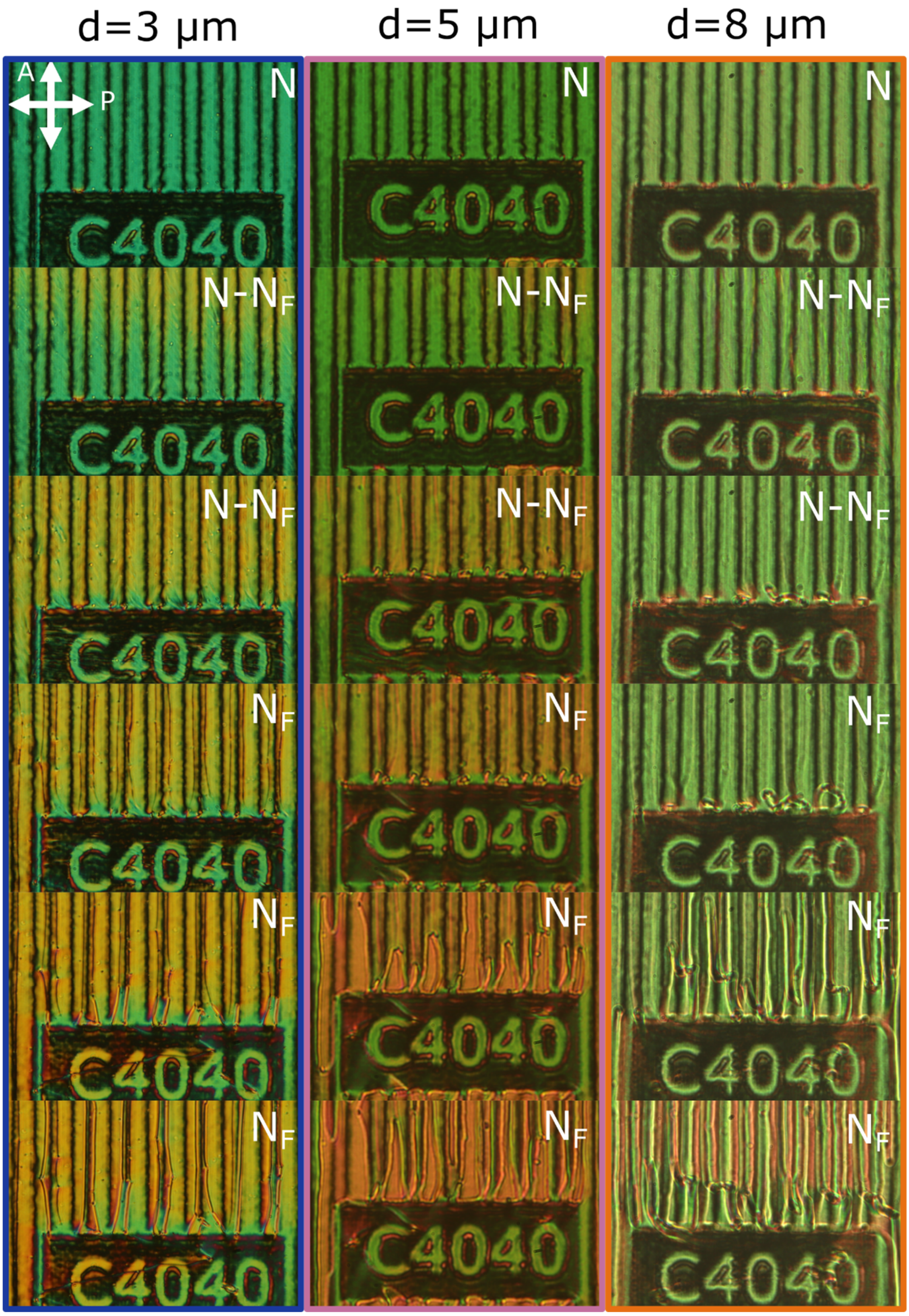

Supplementary Fig.8. Details of the N-N_F transition in RM734 in the splay pattern $\vartheta = \vartheta_0 \sin(2\pi x/P)$ with $\vartheta_0 = 40°$ and $P = 40\ \mu m$ for three different cell thicknesses d= 3, 5 and 8 µm. Last two rows show the propagation of the structural relaxation. For d=3 µm, final structure resembles that of the intermediate state with the surfaces constraining the appearance of twist. In the case of the thicker cells with d= 5 and 8 µm clear differences can be observed, with the final structural relaxation involving the appearance of more complex structures.

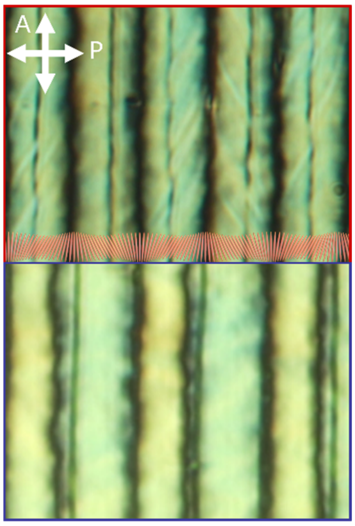

Supplementary Fig.9. Different positions of the disclination lines with respect to the photopatterned splay structure as observed in 3.06 μm cell.

Supplementary Note IV - Dtmm transmission spectra simulations

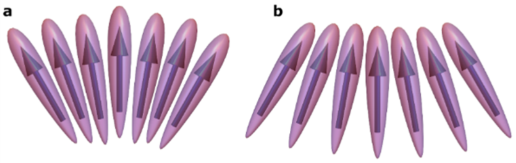

Supplementary Fig.10 For a polar nematic phase, made of asymmetric molecules, due to minimization of excluded volume, polarization and deformation are coupled. That is, there is a favourable splay (a) and a non favourable splay (b) with respect to polarization direction.

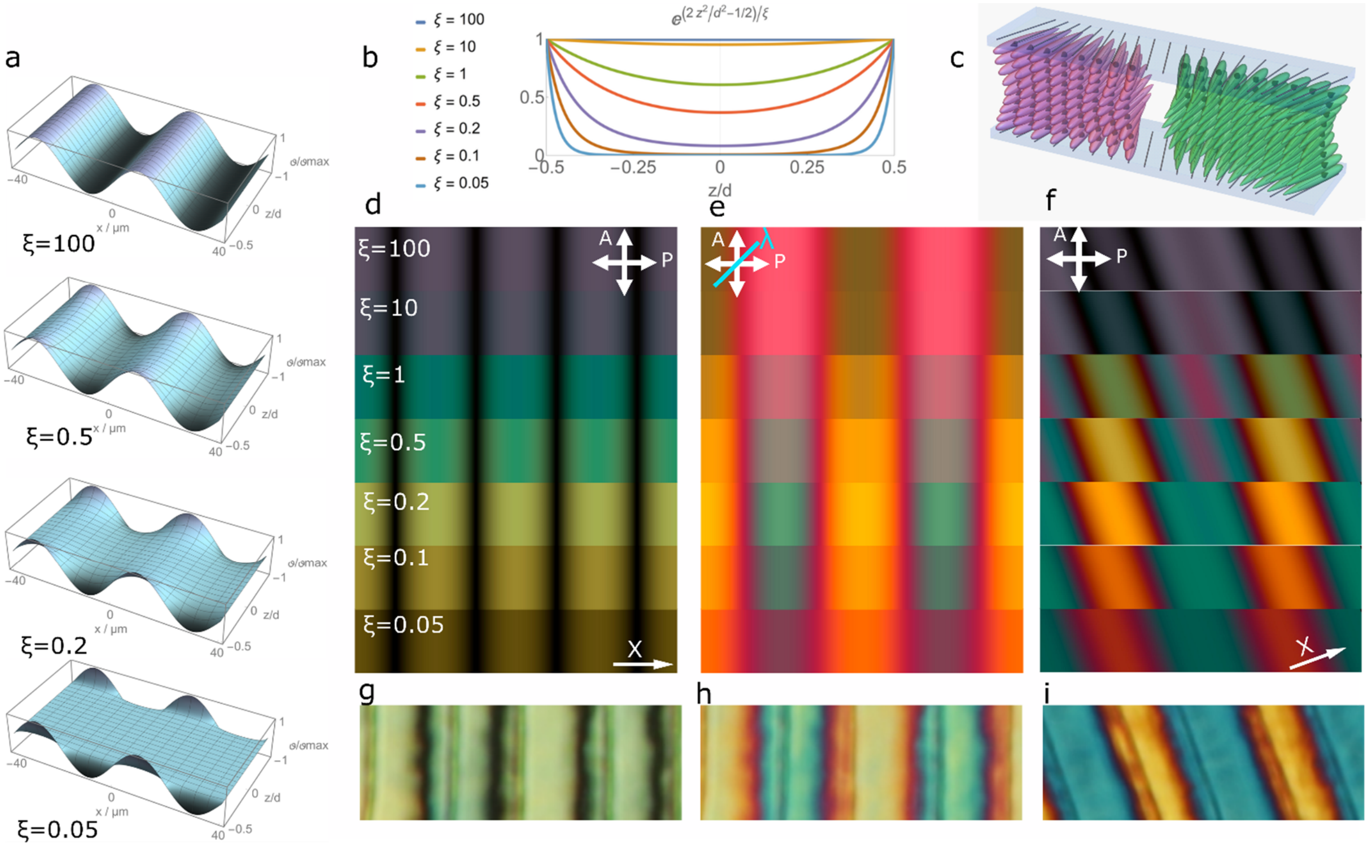

Supplementary Fig.11. Effect of twist thickness on calculated transmitted intensity patterns by dtmm considering a splay pattern $\vartheta_{surf} = \vartheta_0 \sin\left(\frac{2\pi x}{P}\right)$with maximum angle $\vartheta_0 = 40°$, periodicity $P = 40\ \mu m$, $\Delta n = 0.19$, where n_o is taken to be 1.52 and the thickness of the cell is 3.06 µm. The most clear evidence showing that the inscribed surface splay decreases in amplitude towards the centre of the cell arises from the optical transmission texture obtained when rotating the sample. While for a uniform splay structure across the cell ($\xi = 100$), no alternating colours are expected, experiments clearly show blue and yellow areas. Such behaviour, and complementary conditions, can be well simulated with dtmm considering a structure in which the director unsplays towards the cell centre according to $\vartheta = \vartheta_{surf} e^{(2z^2/d^2-1/2)/\xi}$, with $\xi = 0.2$ and being $\vartheta = 0°$ along the periodic lines. Corresponding dtmm simulations at different conditions can be found in Supplementary Fig.15. (a) Angle profile across the cell thickness and two splay periods for different ξ values. (b) Profile of $e^{(2z^2/d^2-1/2)/\xi}$ across the cell thickness for the ξ values used later in (d-f). (c) Schematic representation of the director structure inside the confining cell. (d-f) Dtmm transmission spectra simulations for different ξ values for a sample with the lines aligned with respect the crossed polarizers (d), with an additional lambda plate inserted at 45° as indicated by the blue line (e) and for the sample rotated 20 degrees with respect to the crossed polarizers (f). (g-i) Experimental POM images of the periodic splay pattern obtained for DIO in the same conditions as those simulated.

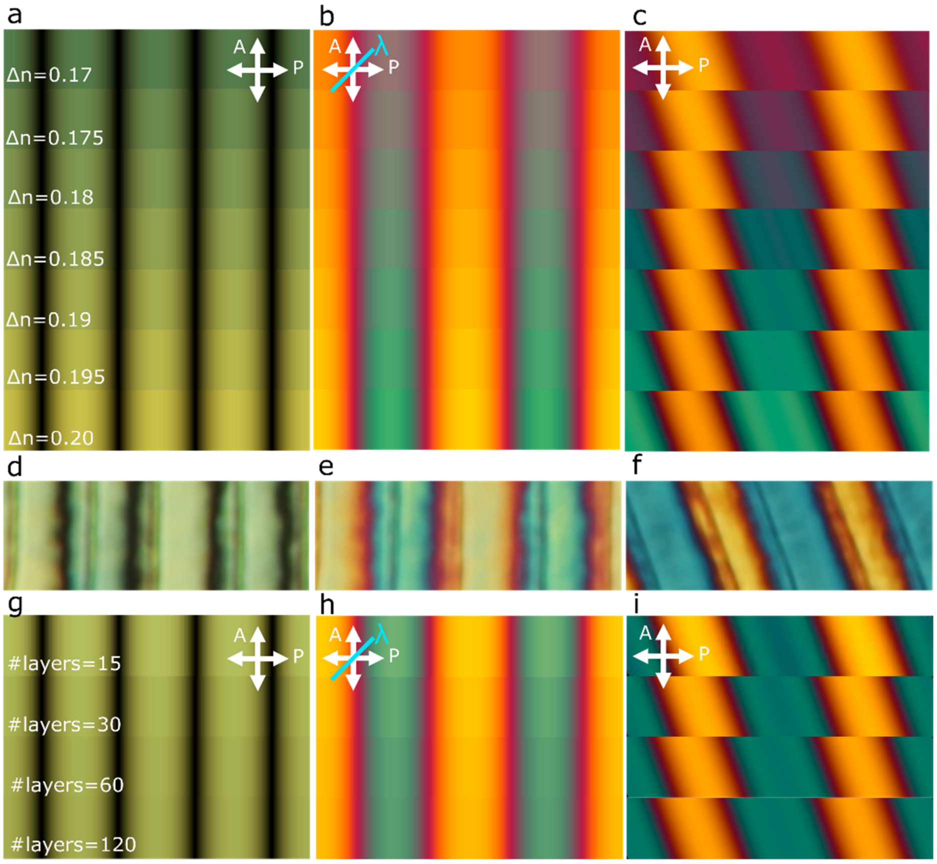

Supplementary Fig.12. (a-c) Calculated transmitted intensity splay patterns for a maximum splay angle of 40° and $\xi = 0.2$ for different birefringence values. The ordinary refractive index value n_o is taken to be 1.52 and the thickness of the cell is 3.06 μm. Dtmm simulations for three different conditions: (a) pattern aligned with crossed polarizers, (b) with a lambda plate inserted at 45° as indicated by the blue line and (c) for the sample rotated 20 degrees with respect to the crossed polarizers. (d-f) Show the corresponding POM images of the periodic patterns in DIO. (g-i) Dtmm simulations for $\Delta n = 0.19$ considering different number of discretization layers for a 3.06 μm cell.

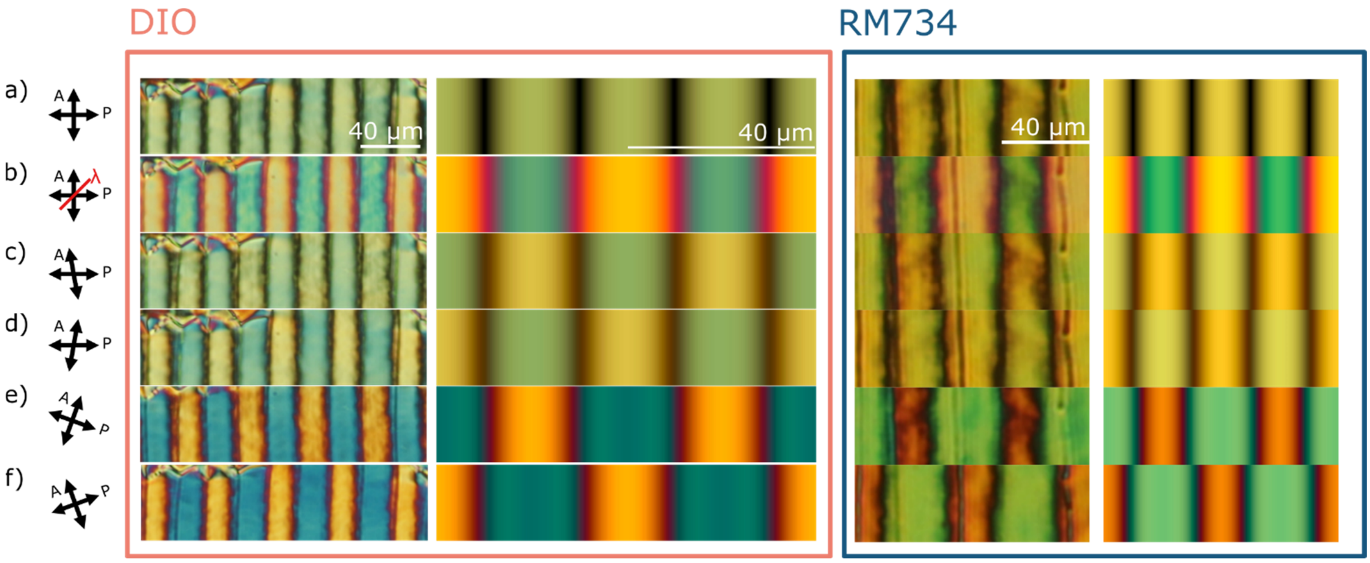

Supplementary Fig.13. DIO & RM734: Comparison of POM images and dtmm simulations for all the studied geometries for the splay pattern with $\vartheta_0 = 40°$, $\xi = 0.2$ and P= $40\ \mu m$ in a 3.06 μm cell. Dtmm simulations were performed considering $\Delta n = 0.19$ for DIO and $\Delta n = 0.21$ for RM734, where n_o is taken to be 1.52. a) Crossed polarizers along the pattern, b) with lambda plate, c&d) uncrossing analyser in opposite directions and e&f) rotating the sample in opposite directions.

Supplementary Note V – Polarization guiding structures

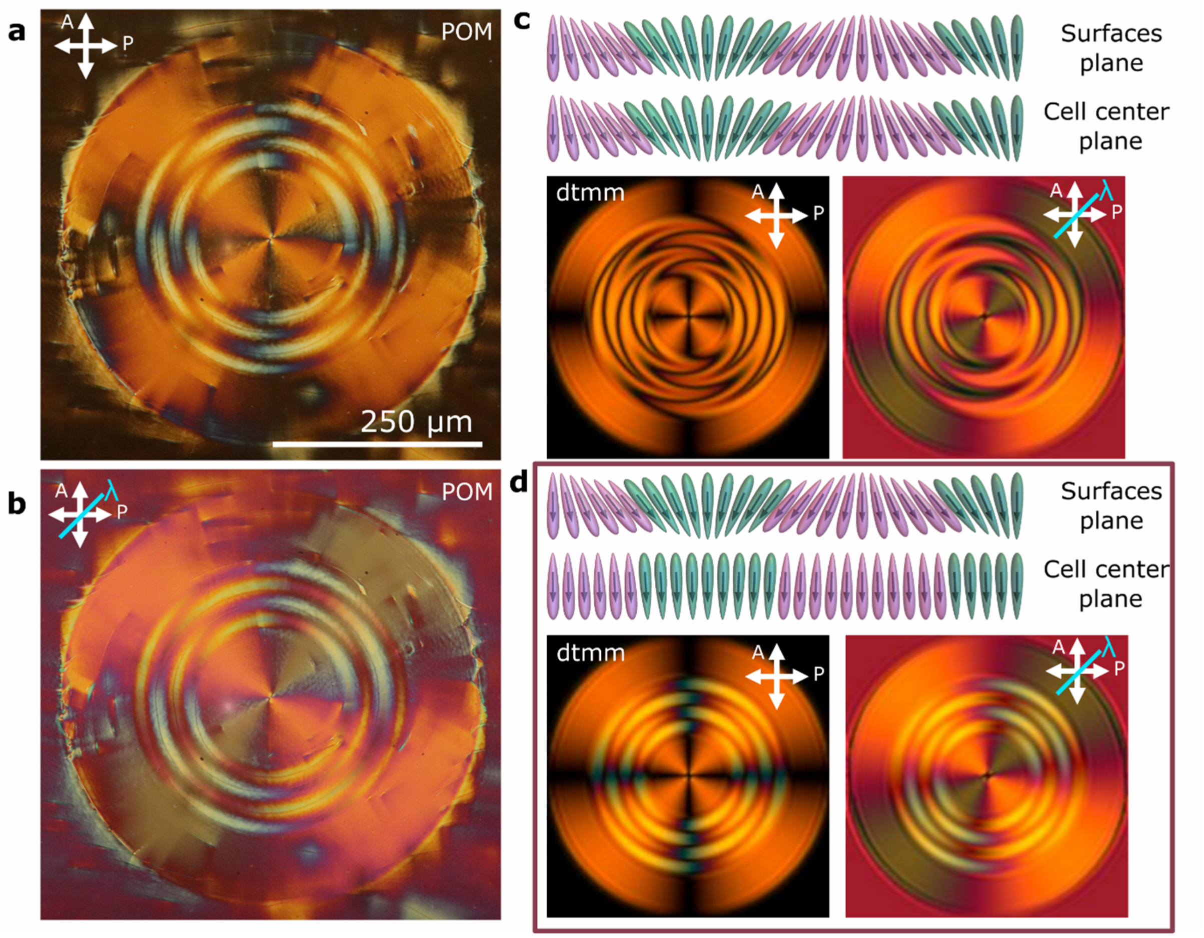

Supplementary Fig.14. Comparison of POM observations (a&b) and dtmm transmission spectra simulations (c&d) for DIO in a structured bend circle in which several splay lines are tangentially embedded. POM texture a) under crossed polarizers and b) with a full lambda plate inserted at 45 degrees as indicated by the blue line. Dtmm simulations were performed considered a uniform splay across the cell thickness d (c) and (d) a structure in which azimuthal angle varies as $\vartheta = \vartheta_{surf}e^{(2z^2/d^2-1/2)/\xi}$ with $\xi = 0.2$ in the splay regions and considering $\Delta n = 0.19$.

Supplementary Note VI – Model for the structure of the splay patterns:

In an apolar nematic liquid crystal, the structure of the director field is determined by a balance of elastic, electric/magnetic and surface torques[5]. In a ferroelectric NLC, the field contribution includes also so called depolarization field, which is electric field caused by the ferroelectric body itself. To assess the stability of the structures deduced from POM in the case of periodic splay pattern, we used a simplified model.

First, the local field **E** was calculated using the following expression for the director field **n**(**r**) within one half of a periodic splay pattern:

$$\mathbf{n}(x,z) = (\sin\vartheta(x,z), \cos\vartheta(x,z)\cos\varphi(x,z), \cos\vartheta(x,z)\sin\varphi(x,z)) \tag{1}$$

$$\mathbf{P}_s = P_0\mathbf{n} \tag{2}$$

$$\vartheta_i(x,z) = A_0 \sin\frac{k_x x}{2} e^{(2z^2-0.5d^2)/(\xi d^2)}\left(1 - e^{(2x^2-0.5L^2)/(0.05L^2)}\right), \tag{3}$$

$$\varphi_i(x,z) = 0 \tag{4}$$

where *d* is the thickness for the LC cell, $\xi = 0.2$ as descried in the manuscript, $k_x = \frac{2\pi}{L}$, and *L* a half of the splay period *P*. In the other half of the pattern $\vartheta_1(x,z) = \vartheta(x,z) + \pi$ and $\varphi_1(x,z) = \pi - \varphi(x,z)$. At the cell surface boundaries $(z = \pm d/2)$, the orientation of **n** is prescribed by the photo-pattern with $\vartheta_s(x) = A_0 \sin\frac{k_x x}{2}\left(1 - e^{(2x^2-0.5L^2)/(0.05L^2)}\right)$, with the last term being discussed below. To simplify calculations, it was assumed the dielectric tensor is isotropic, $\boldsymbol{\varepsilon} = \varepsilon\mathbf{I}$.

In the case when there are no free ions, the local field is the depolarization field, which can be calculated using bound charges for being its source. The bound charge has two contributions, volume charge $\rho_b = -\nabla\cdot\mathbf{P}_s$, and surface charge $\sigma_b = \boldsymbol{\nu}\cdot\mathbf{P}_s$, where **ν** is a vector normal to the surface of the ferroelectric. The Eq.3 differs from the assumed structure in dtmm simulations in the last factor, which is used to transform surface bound charge in the edges of the splay to a smooth charge distribution within a thin layer ($\approx 0.025L$) at the boundary of our calculation box, so that $\sigma_b = 0$. That is, cell surface boundaries are considered as prescribed by photopatterning everywhere expect in a thin layer ($\approx 0.025L$) around $x = \pm L/2$ where the splay changes sign. The electrostatic potential $\Phi(\mathbf{r})$ is calculated using the Poisson equation

$$\nabla^2\Phi_{dep} = -\frac{\rho_b}{\varepsilon\varepsilon_0} \tag{5}$$

in a box $(-P/4 \le x \le P/4, -d/2 \le z \le d/2)$, which is periodic in the *x*-direction and finite in the *z*-direction. Because ρ_b is the same in both halves of the splay pattern, Eq.(5) needs to be solved only for half of the pattern.

In a realistic case, there are free ions present in the material, which screen the depolarization field. Assuming the free charge density follows Boltzmann distribution, $\rho^{\pm} = \pm e n_0 \mathrm{Exp}\left(\mp e\Phi/(k_B T)\right)$, and positive and negative ions have the absolute charge, i.e., they carry a charge $e = \pm Z e_0$, (where e_0 is the elementary charge, *Z* positive integer, and the number density n_0 is the same for positive and negative free ions), the electrostatic potential can be calculated using Poisson-Boltzmann equation

$$\nabla^2\Phi_n = \beta^2\sinh\Phi_n - \rho_{b,n} \tag{6}$$

Here, $\Phi_n = e\Phi/k_B T$. If $\Phi_n < 1$, as an approximation, a linearized Poisson-Boltzmann equation

$$\nabla^2\Phi_n = \beta^2\Phi_n - \rho_{b,n} \tag{7}$$

can be used to evaluate local electric fields. We used such a normalization that $\beta^2 = (2n_0 e)/(P_0 k_x A_0)$, $\rho_{b,n} = \rho_b/(P_0 k_x A_0)$, and the length is normalized to $\xi_b = \sqrt{(\varepsilon\varepsilon_0 k_B T)/(P_0 k_x A_0)}$. In this normalization, the coefficient $\beta = \xi_b/\lambda_D$, where $\lambda_D = \sqrt{(\varepsilon\varepsilon_0 k_B T)/(2n_0 e^2)}$ is the Debye length. The linearized Poisson-Boltzmann equation is valid provided that the screening length λ_D is smaller than the features of the system and ξ_b.

Two cases of boundary conditions were considered, (a) the material is in contact with grounded electrodes:

$$\Phi\left(x, \pm\frac{d}{2}\right) = 0, \tag{8}$$

or (b) with glass with the dielectric constant ε_g:

$$\frac{\partial\Phi}{\partial x}\left(x, \pm\frac{d}{2}\right) = \frac{\partial\Phi_{glass}}{\partial x}\left(x, \pm\frac{d}{2}\right) \tag{9}$$

$$\varepsilon\frac{\partial\Phi}{\partial z}\left(x, \pm\frac{d}{2}\right) = \varepsilon_g\frac{\partial\Phi_{glass}}{\partial z}\left(x, \pm\frac{d}{2}\right), \tag{10}$$

where Φ_{glass} is the electrostatic potential in the glass. ρ_b was approximated with a finite Fourier series, and Eq.7. was solved numerically in Fourier space. The field was calculated as $\mathbf{E} = -\nabla\Phi$.

In Supplementary Fig.15 and Fig.16, the comparison of the local field is shown for the two boundary conditions and different ion concentrations for the initial structure and ε =100 or 1000 respectively. In the calculations, the following values were used: $P_0 = 0.05\frac{\mathrm{As}}{\mathrm{m}^2}$, $d = 3\ \mu\mathrm{m}$, $L = 20\ \mu\mathrm{m}$, $A_0 = 40°$, ε =100 or 1000, $\varepsilon_0 = 8.85 \cdot 10^{-12}\frac{\mathrm{As}}{\mathrm{Vm}}$, $k_B T = 4 \cdot 10^{-21}$J, Z=1, $e_0 = 1.6 \cdot 10^{-19}$As and β^2 =0, 1 and 10, which correspond to ion concentrations of $n_0 = 0$ m^{-3}, $1.7\cdot10^{22}$m^{-3} and $1.7\cdot10^{23}$m^{-3}. The comparison of the order of magnitude of the electrostatic energy density $1/2\ \mathbf{P}\cdot\mathbf{E}$ with twist elastic energy $K_2/(0.1d)^2 \sim 20\ \mathrm{J/m^3}$ shows that in all cases, the local fields are too large for the structure to be stable. (Here, K_2 is the twist elastic constant.) This means that the electric field torque acting on **P** is much larger than the nematic elastic torque, and, consequently, the structure will relax towards stable structure determined by the minimum of the free energy. To assess the difference between the initial and relaxed structure, a simplified model for free energy was used.

In general, FNLC can be described by two coupled order parameters, a nematic quadrupolar, i.e., tensor $\mathbf{Q} = S(\mathbf{n}\otimes\mathbf{n})$, and electric polarization vector **P**[6]. Here, S is the scalar order parameter and **n** the director (with the symmetry $\mathbf{n} \equiv -\mathbf{n}$), which denotes direction of the average orientation of the molecules in the nematic phase[5]. In the model, we made the following assumptions: (i) S is constant, so the nematic order can be described only by **n**; (ii) $\mathbf{P} = \mathbf{P}_s + \varepsilon_0(\boldsymbol{\varepsilon} - \mathbf{I})\mathbf{E}$, where $\mathbf{P}_s = P_0\mathbf{n}$; (iii) P_0 is constant; (iv) induced polarization anisotropy is neglected and dielectric tensor is thus taken as isotropic, $\boldsymbol{\varepsilon} = \varepsilon\mathbf{I}$; and (v) the orientation of the director at the surface is the same as prescribed by photo-patterning, that is so-called strong anchoring boundary condition. By assuming P_0 is constant, we neglected the part of changes in the polarization value due to the flexoelectric effect. The values of spontaneous polarization in the ferroelectric phase are large, so it is expected that the small change in its value due to the flexoelectricity can be neglected in our simplified approach. This assumption is also supported by the results of the SHG microscopy, where in the N_F we did not notice significant changes in SHG intensity in the parts of the sample with more splay.

Here, it has to be noted, that this simplified model is not suitable for the description of topological defects, around which neither S nor P_0 is expected to be constant. The model is only used to assess whether this simple approach can explain the observed structures away from the defects. Additionally, while the effective value of dielectric constant measured by dielectric spectroscopy is large, i.e. of the order of 10000 (which mainly comes from the reorientation of $\mathbf{P}_s$)[6], the value of ε as defined above only accounts for induced polarization and is expected to be of the order 100 – 1000. The value is larger close to the ferroelectric phase transition and it decreases with the temperature, i.e., when the system is deeper in the ferroelectric nematic phase.

Stable structures can be found by minimization of the Landau-de Gennes type of free energy functional. In the general case when free ions are present, the functional must include also the electrostatic potential[7]. However, if we assume that the dynamics of free charges is much faster than the dynamics of **n**, then during the relaxation of **n**, the electrostatic potential is given by the solution of the Poisson-Boltzmann equation. In such case, the relaxation method can be used to minimize the the part of the Landau-de Gennes free energy that depends on the director orientation, i.e. the relevant part of the Landau-de Gennes type of free energy functional:

$$F_{\mathbf{n}} = \int \left(\tfrac{1}{2}K_1|\mathbf{S}-\mathbf{S}_0|^2 + \tfrac{1}{2}K_2 Tw^2 + \tfrac{1}{2}K_3|\mathbf{B}|^2 - \tfrac{1}{2}P_0\mathbf{n}\cdot\mathbf{E}\right) dV\,. \qquad (11)$$

The relaxation steps were performed with respect to $\vartheta(x,z)$ and $\varphi(x,z)$. At each step, **E** was recalculated using Eq.7. Here, K_i (i = 1,2,3) splay, twist, and bend elastic constants with corresponding deformations $\mathbf{S} = \nabla\cdot\mathbf{n}, Tw = \mathbf{n}\cdot(\nabla\times\mathbf{n})$, $\mathbf{B} = \mathbf{n}\times(\nabla\times\mathbf{n})$, and $\mathbf{E} = -\nabla\Phi$. The flexoelectric term is included in the first term, where $\mathbf{S_0} = \gamma\mathbf{P}\cdot /K_1$ is the ideal splay curvature, which would minimize the splay elastic energy. The sign of $\mathbf{S_0}$ determines the preferred direction of **P** when splay deformation is present in the system. The ideal splay curvature which would minimize the splay elastic energy, is $\mathbf{n}\cdot\mathbf{S}_0$. However, because of the assumptions (ii) and (iii) described above, $\boldsymbol{S}_0$ does not enter the local relaxation equations for $\vartheta(x,z)$ and $\varphi(x,z)$. In the calculations the following values of elastic constants were used: $K_1 = K_3 = 20$ pN, $K_2 = 2$ pN. (Note: If the dielectric tensor is taken anisotropic, i.e. $\boldsymbol{\varepsilon} = \varepsilon_\perp\mathbf{I} + \Delta\varepsilon(\mathbf{n}\otimes\mathbf{n})$, there would be an additional term $-\frac{1}{2}\Delta\varepsilon\varepsilon_0(\mathbf{n}\cdot\mathbf{E})^2$, which is orders of magnitude smaller than the term $-\frac{1}{2}P_0\mathbf{n}\cdot\mathbf{E}$, due to the large polarization values ($\frac{P_0}{\varepsilon_0} \sim 5\cdot\ 10^9$ V/m). So from this point of view, neglecting the dielectric anisotropy is justified.)

Supplementary Fig.17 shows the local field in the case of two examples of relaxed structures. At the edges of the pattern, where defects are observed in the experiment, the local fields and deformations are large, counteracting each other. As already discussed, in this region, our simplified model is not expected to yield realistic results and we will not discuss it further. If the density of ions is sufficient ($\beta^2 \gtrsim 1$), the free ions screen the part of the depolarization field originating from the polarization in the part of the sample which is more than a few screening lengths away from a given point. Therefore, the structure at the edge has little or no effect on the structure in the middle of the pattern. In Supplementary Fig.18, the local fields away from the edges in the structure after minimization are compared with the initial one for the case screening length is 20 nm ($\beta^2 = 10, \varepsilon = 100$) and in Supplementary Fig.19 the corresponding $\vartheta(x,z)$ and $\phi(x,z)$ values of the structure across the region are shown. Although the structure differs very little from the initial one, the decrease of the depolarization field is substantial, i.e., more than an order of magnitude (Supplementary Fig.18). Most notable is the appearance of out-of-plane splay deformation, which causes such a redistribution of bound and free charges (Supplementary Fig.20) that the field is reduced. It has to be noted here that the region as a whole is neutral, so the sum of bound charges and the sum of free charges are both zero. This means that independently of what the structure around the defects at the edge of the splay is, the total bound charge there is the same. Its value is equal to minus the sum of the charge in the middle of the splay structure, so the total bound charge is zero.

As shown by SHG-I, disclination lines separate regions with opposite polarization direction. As shown in Supplementary Fig.21, although appearing at the edge of the splay patterns, in some occasions they have also been observed towards the center of the splay region. This could be attributed to slight asymmetries of the photopatterned surface splay structure, which would provoke asymmetries in the otherwise evenly distributed charge density expected for perfect splay structure shown in Supplementary Fig.21. Such structure asymmetry would then cause a shift of the polarization reversal region towards the center of the splay, which carries opposite electric charge.

Due to the polarity of the phase, topological charge of the disclination lines should be an integer. Additionally, in order to avoid electrical charge in the core, twist deformations are the most favourable, and thus, a possible structure of such lines could be a twist-like disclination with topological charge ±1 as shown in Supplementary Fig.21.b. When embedded in a non-splayed uniform background, the overall structure is electrically neutral. However, when embedded in a splayed background, it carries a charge as discussed above.

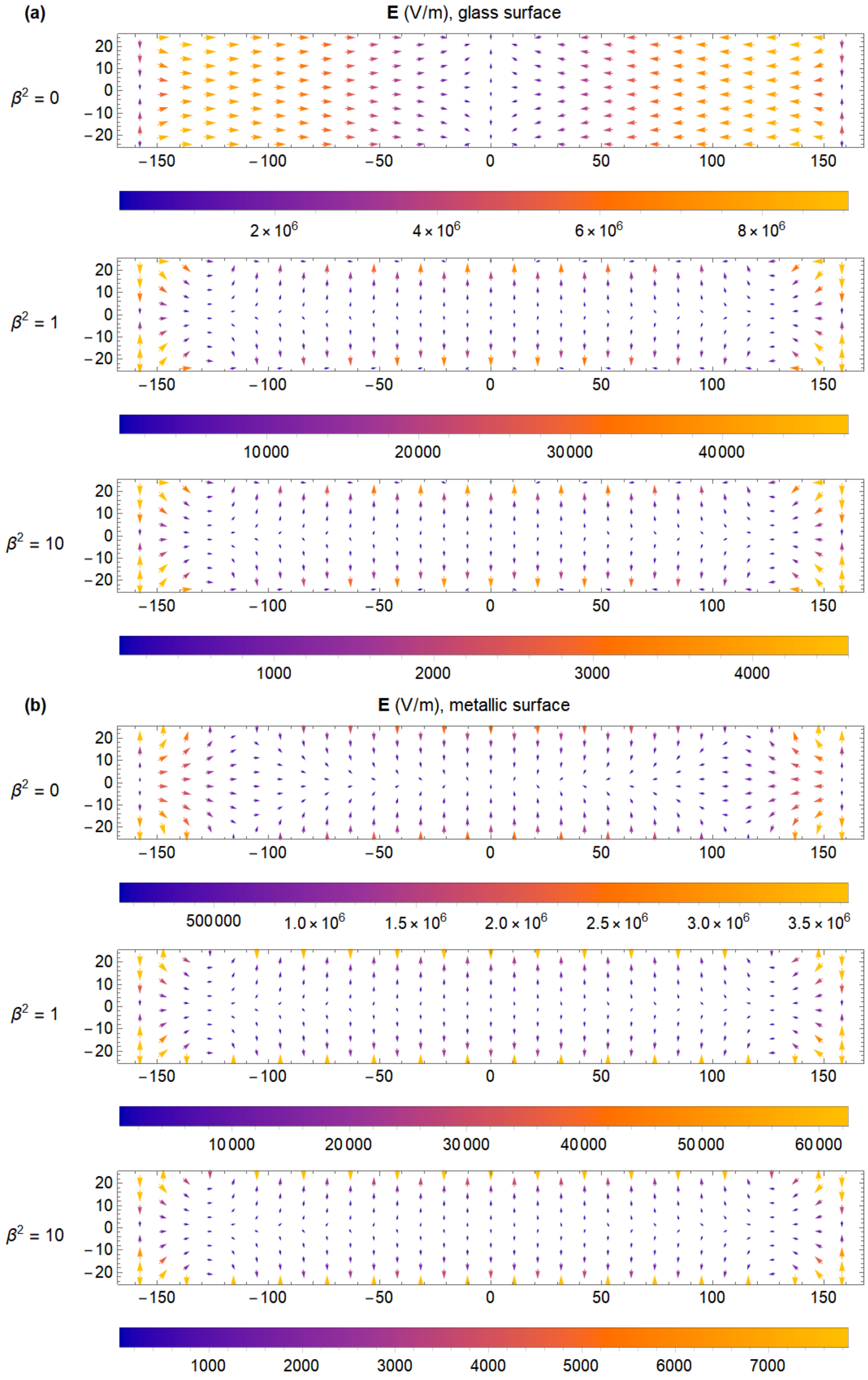

Supplementary Fig.15: xz- cross section of the local field E in units of V/m calculated for the structure given by Eqs. (1) –(3) for (a) glass surface and (b) grounded metal surfaces for ε = 100, and β as marked. The x and z coordinates are is given in units of $\xi_b = 63$ nm.

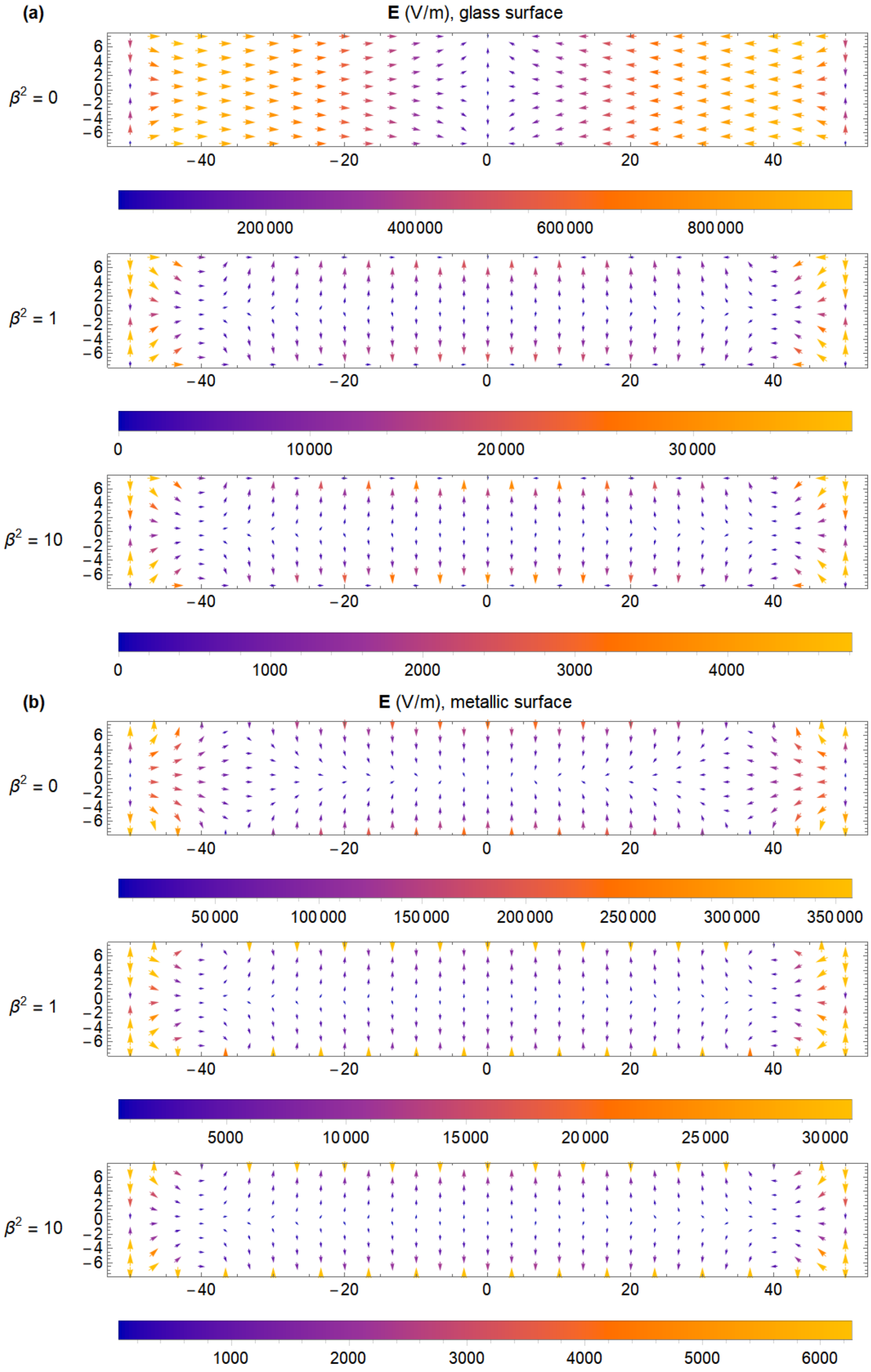

Supplementary Fig.16: xz- cross section of the local field E in units of V/m calculated for the structure given by Eqs. (1) – (3) for (a) glass surface and (b) grounded metal surfaces for ε = 1000, and β as marked. The x and z coordinates are given in units of $\xi_b = 200$ nm. For ε = 1000 and $\beta^2 = 1$, $\lambda_D = 200$ nm and ϕ_n exceeds 1 only at the edges of the splay $\sim \pm L/2$, where it reaches the value of 1.5. Comparison of the solution of general Poisson-Boltzmann equation with linearized equation, shows that this accounts for an error of around 10% in that region.

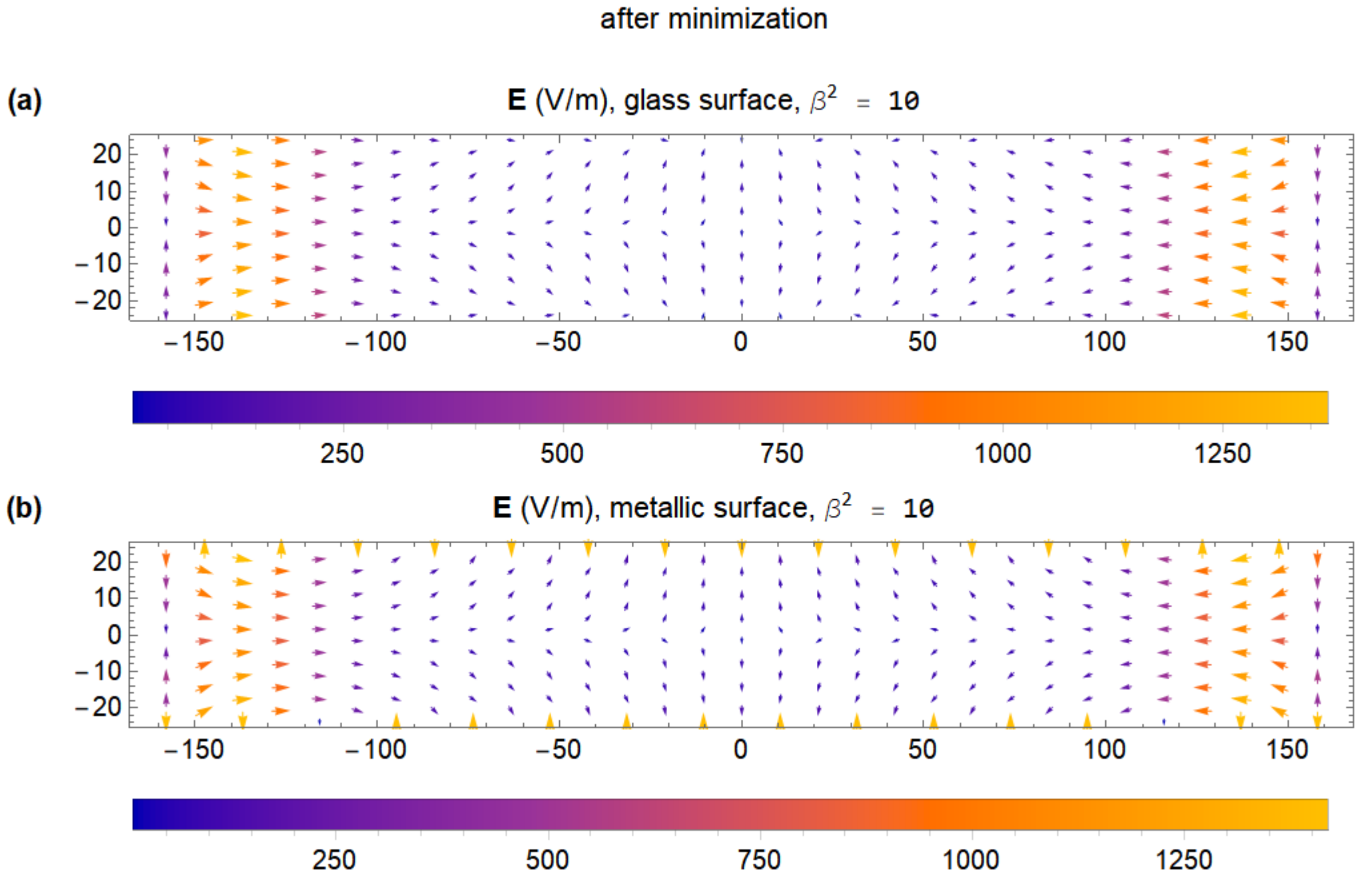

Supplementary Fig.17: xz- cross section of the local field for the structure after relaxation for $\beta^2 = 10$ and $\varepsilon = 100$ for (a) glass surface and (b) grounded metal surfaces. The x and z coordinates are is given in units of $\xi_b = 63$ nm.

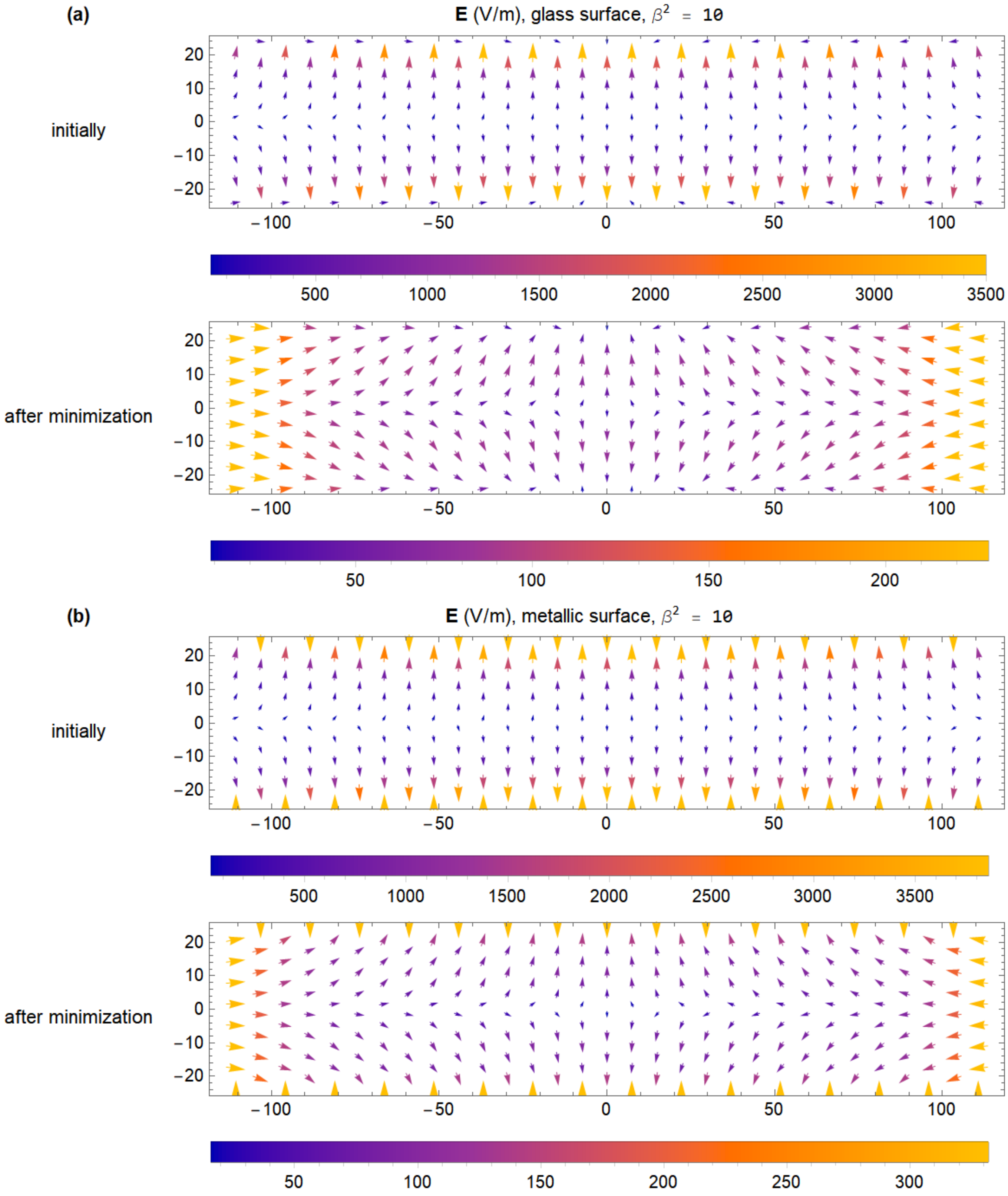

Supplementary Fig.18: Comparison of the local field for initial structure and the structure after relaxation in the central region $(-0.7\ L/2 < x < 0.7\,L/2)$ for $\beta^2 = 10$ and $\varepsilon = 100$ for (a) glass surface and (b) grounded metal surfaces. The x and z coordinates are is given in units of $\xi_b = 63$ nm.

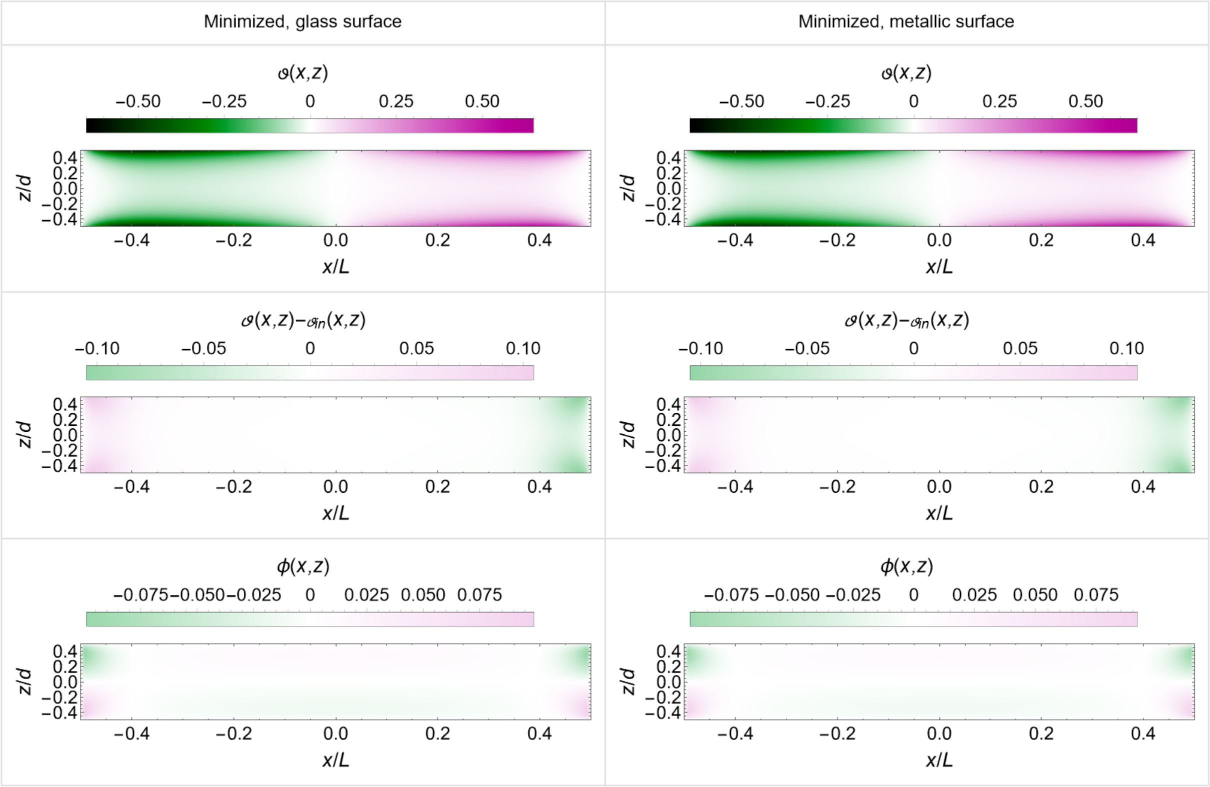

Supplementary Fig.19: Comparison of the structure before and after relaxation in the central region for both types of the surface for $\beta^2 = 10$ and $\varepsilon = 100$. The initial structure is given by angles $\vartheta_{in}(x,z)$ and $\phi_{in}(x,z) = 0$ as described in Eq.3 and Eq.4.

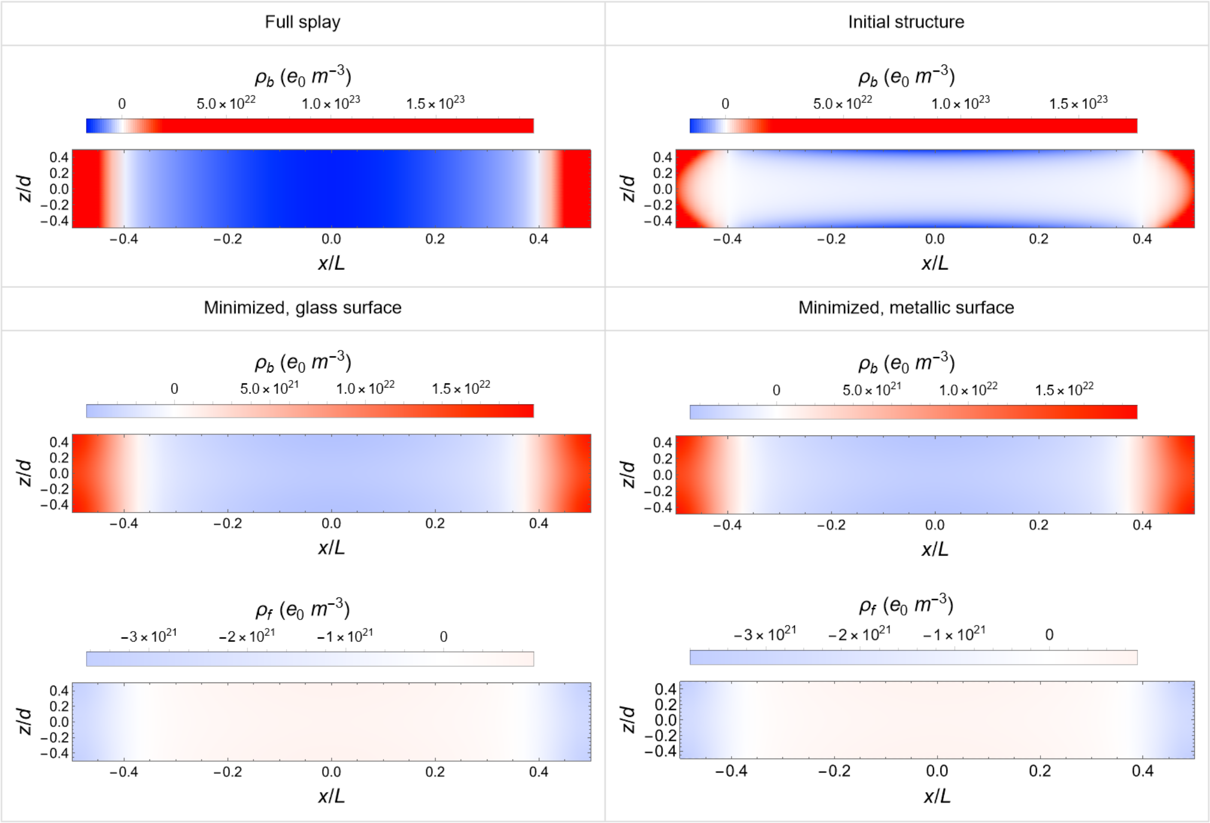

Supplementary Fig.20: Comparison of bound charge density ρ_b for fully splayed structure (i.e. splay deformation independent of z), the initial unsplayed structure and relaxed structures for both boundary conditions. For relaxed structures also free charge density ρ_f is shown.

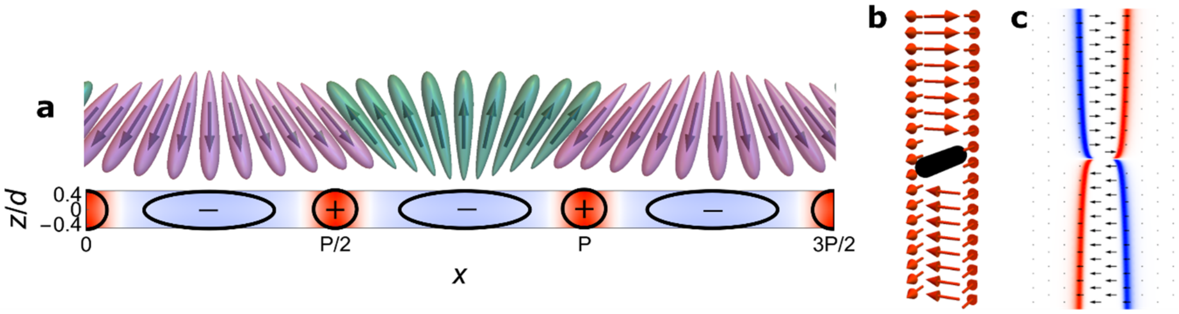

Supplementary Fig.21. Twist-like disclination lines representation. (a) Schematics of the bound charge density variation in splay patterns and (b) schematic of proposed twist polarization deformation around disclination lines. Front view, together with the calculated bound charge density highlighted in red and blue is depicted in (a). Similarly, the bound charge density of the structure in (b) can be observed in (c).

Supplementary Note VII – Interferograms fitting function

In the SHG interferometry setup, the recorded intensity can be expressed as:

$$I = \left(A_{sample}\sin(kz) + A_{referemce}\sin(kz+\delta)\right)^2 \quad (12)$$

The phase between the SHG signal of the sample and that of the reference is varied by inserting a glass slide (thickness d) and varying the incidence angle (ϕ) and then δ can be written as:

$$\delta = \delta_0 + \frac{kd}{\sqrt{1-\frac{\sin^2(\phi)}{n^2}}} \quad (13)$$

where $k = 2\pi n/\lambda$. Interferometry sets of data have then been fitted to

$$I = A_{sample}^2\left(1 + A\sin\left(\delta_0 + \frac{\frac{2\pi nd}{\lambda}}{\sqrt{1-\frac{\sin^2(\phi-\phi_0)}{n^2}}}\right)\right)^2 \quad (14)$$

with λ =780/2 nm and $A = A_{reference}/A_{sample}$. Reasonable values of n and d for the inserted glass plate were obtained from a set of fits to be n=1.516 and d=1.046 mm and subsequently fixed for the rest of data sets.

References

1. Li, J. *et al.* Development of ferroelectric nematic fluids with giant-ε dielectricity and nonlinear optical properties. *Science Advances* **7**, eabf5047 (2021).
2. Mandle, R. J., Cowling, S. J. & Goodby, J. W. Rational Design of Rod-Like Liquid Crystals Exhibiting Two Nematic Phases. *Chem. Eur. J.* **23**, 14554–14562 (2017).
3. Nys, I., Berteloot, B., Beeckman, J. & Neyts, K. Nematic Liquid Crystal Disclination Lines Driven by A Photoaligned Defect Grid. *Advanced Optical Materials* **10**, 2101626 (2022).
4. Lovšin, M. & et al. *in preparation* (2023).
5. de Gennes, P. G. & Prost, J. *The Physics of Liquid Crystals*. (Clarendon Press, 1995).
6. Sebastián, N. *et al.* Ferroelectric-Ferroelastic Phase Transition in a Nematic Liquid Crystal. *Phys. Rev. Lett.* **124**, 037801 (2020).
7. Everts, J. C. & Ravnik, M. Ionically Charged Topological Defects in Nematic Fluids. *Phys. Rev. X* **11**, 011054 (2021).
