# Supplementary material for: Polarization patterning in ferroelectric nematic liquids via flexoelectric coupling
Source: Nat Commun. 2023 May 25;14:3029. doi: 10.1038/s41467-023-38749-2 (PMC10213025; doi:10.1038/s41467-023-38749-2)
Supplement: Supplementary file 3 — Description of Supplementary Information [file 41467_2023_38749_MOESM3_ESM.pdf]

Inventory of Supplementary Information

File Name: NSebastian_etal_SupplementaryInformation.pdf

Description: supplementary information file

File Name: Supplementary Movie 1.mp4:

Description: Supplementary Movie 1 shows, for DIO, the transition between the antiferroelectric splay nematic phase and the ferroelectric nematic phase when confined in a photopatterned 3 µm cell in which the surfaces impose a periodic splayed structure with maximum splay angle of 40 degrees and splay period P= 40 µm. Pretransitional behaviour is characterized by a clearly visible stripe texture, with stripes following the photopatterned structure. At the transition disclination lines are formed uniformly along the pattern in those regions in which splay changes sign and progressively the texture between lines becomes uniform. Few degrees below the transition there is a final structural relaxation, characterized by the propagation of a final deformation along the disclination lines, forming domain walls. Such deformation involves charges that slightly reorient polarization around it, evidenced by the slight change in transmitted spectra. Snapshots together with the transition as observed with SHG-M are shown in Supplementary Fig. 8.
